# Genomic-scale Analysis of Bacterial Gene and Protein Expression in the Host

**DOI:** 10.3201/eid1008.031036

**Published:** 2004-08

**Authors:** John D. Boyce, Paul A. Cullen, Ben Adler

**Affiliations:** * Monash University, Melbourne, Victoria, Australia

**Keywords:** DNA microarray, Proteomics, Host-parasite interaction, Gene expression

## Abstract

The developing complementary technologies of DNA microarrays and proteomics are allowing the response of bacterial pathogens to different environments to be probed at the whole genome level. Although using these technologies to analyze pathogens within a host is still in its infancy, initial studies indicate that these technologies will be valuable tools for understanding how the pathogen reacts to the in vivo microenvironment. Some bacterial pathogens have been shown to substantially modify their surface components in response to the host immune system and modify their energy metabolism and transport pathways to allow efficient growth within the host. Further detailed analyses of these responses will increase understanding of the molecular mechanisms of pathogenesis, identify new bacterial virulence factors, and aid in the design of new vaccines.

## Abstract 2

DNA microarrays and proteomics are used to study bacterial gene and protein expression during infections.

How do bacteria respond to the host environment during an infection? Bacterial pathogens must be able to gain access to, persist in, and replicate in normally privileged sites within a host. Moreover, they must produce certain factors that result in a level of host damage that perturbs homeostasis. Thus, pathogens must have specific mechanisms for mediating colonization, avoiding the host's immune system, and acquiring necessary nutrients. They must also produce factors that result (directly or indirectly) in host damage. Because the environment encountered within a living host will be quite different from the external environment, pathogens must be able to regulate the necessary genes in coordination as they move from the environment to the host and from one host niche to another.

The primary aim of investigating bacterial pathogenesis is to understand the way that pathogens interact with the host to cause disease. Central to this investigation is an understanding of what gene products are required and expressed during a natural infection and how this expression changes over time (from initial colonization to causation of disease and spread of the pathogen to new hosts) and space (in different cells or tissues within the host). We thus endeavor to understand how the pathogen adapts to the host microenvironment, what selective pressures are acting on the pathogen in each microenvironment, what bacterial factors are responsible for the host damage, and how the immune system is evaded. Although analyses that give information on the expression of a few genes provide insight and have been responsible for a large proportion of the bacterial pathogenesis literature currently available, our ultimate goal is to understand expression changes across the whole genome. The additional information generated by whole genome studies goes far beyond that derived by characterizing in isolation more genes and gene products, because analysis of the whole genome allows complete regulatory networks to be identified and characterized. These results cannot be achieved with a "one-gene-at-a-time" approach. Whole genome studies could be considered as an exponential and synergistic advance rather than a linear progression.

The host-pathogen interactions that define a disease are clearly complex, and, in many cases, the study of these interactions is limited by the lack of a suitable animal model. However, we now have a number of methods that allow identification of genes critical for survival in a host as well as methods that allow direct measurement of gene expression during interaction with a host. Two of these methods, signature-tagged mutagenesis and in vivo expression technology, do not directly measure gene expression and do not allow true genomic-scale analysis, but they have been devised to identify genes necessary for pathogens during real infections. Excellent reviews on these techniques are available (1,2), and they will not be discussed in this review. A second group of methods, which includes DNA microarrays and proteomics, have advantages that overcome the limitations implicit in signature-tagged mutagenesis and in vivo expression technology, namely, the ability to directly measure expression (gene or protein) levels on a true genome-wide scale, but their application to analysis of bacterial pathogens during real infections is still in its infancy. Another method, real-time reverse transcriptase polymerase chain reaction (RT-PCR) has qualities that bridge those of the other methods, allowing accurate gene expression measurements but on a subgenomic scale; thus, we will not discuss it in this review. However, real time RT-PCR is useful for coping with the low numbers of microorganisms that are often available during infections, and high-throughput whole-genomic scale real-time RT-PCR may become available in the near future. We summarize the current application of DNA microarray and proteomics techniques to the understanding of how bacteria modify their expression profiles within an infected host.

## DNA Microarray Studies

DNA microarrays offer the promise of accurate gene expression measurements for every gene in a genome and allow this expression to be analyzed in response to any environmental variable. However, this huge potential for the understanding of bacterial pathogenesis has not yet been completely realized because of the substantial technical problems associated with accurately measuring bacterial gene expression during real infections. The main problems associated with their use in such situations include the following: the low numbers of bacteria in living tissues during infection, difficulty in purifying the bacteria (and therefore bacterial RNA) from the eukaryotic tissue, potential mRNA instability and possible differential degradation during purification, and the difficulty in finding an appropriate animal model for many diseases. The instability of bacterial mRNA can in part be overcome by using commercially available RNA stabilization reagents such as RNAlater (Qiagen, Hilden, Germany). Additionally, because of the specificity of DNA hybridizations, small amounts of co-purified eukaryotic RNA are unlikely to adversely affect the microarray results. However, this type of transcriptional analysis can currently only be applied to those infections that lead to high titers of infecting organisms in host tissues, since the experiments require at least microgram quantities of RNA. Thus far, no one has accurately measured gene expression throughout an infection, from the initial stage of invasion/colonization through multiplication and tissue spread to the final stages of disease with notable host damage. Such a complete analysis of gene expression remains the "holy grail" of gene expression measurements with regard to bacterial pathogenesis. However, we are beginning to see a number of experiments that provide insights into the way bacteria regulate gene expression at different phases of infection.

The first wave of DNA microarray experiments of relevance to bacterial pathogenesis focused on analyzing bacterial gene expression during growth in vitro under conditions chosen to mimic some aspect of infection. In many cases, the relationship to a specific condition that the bacteria will face during growth in the host is clear, and as the conditions are manipulated in vitro, the test conditions can be tightly controlled. Thus, these studies have allowed a detailed description of bacterial pathogen response to iron limitation (3,4), nutrient limitation (5,6), acidic environments (7,8), low oxygen (9,10), bacterial density (11,12), and biofilm formation (11,12). A number of studies have also analyzed the global effects of transcriptional regulators with the aim of defining complete regulatory networks (13).

Although the in vitro experiments have added substantially to our understanding of gene expression in bacterial pathogens, they can never completely model the conditions that a pathogen encounters in a host during infection. Thus, the second wave of microarray experiments has focused on directly measuring bacterial gene expression during interaction with eukaryotic cells or during growth within the host (Table). Three studies have directly analyzed whole-genome bacterial gene expression during growth in the tissues of a living eukaryotic host (18–20), while a fourth analyzed gene expression in bacteria recently exited from host tissue, the lumen of the bowel (21). All four experiments compared growth in vivo with growth in in vitro laboratory medium. Three of the studies carried out competitive hybridization of in vivo and in vitro RNA to directly compare gene expression in the two sites, whereas the third study compared gene expression levels of both in vivo and in vitro samples to a common reference sample (genomic DNA). Despite the analyses being carried out on different bacterial species (*Vibrio cholerae* and *Pasteurella multocida*), striking similarities between the gene expression changes were seen in all four experiments.

**Table:** Large-scale measurement of bacterial gene or protein expression during real infections or interactions with host cells

Species	Experiment type	Description	Reference
Growth in the host			
*Borrelia burgdorferi*	DNA microarray	Growth in dialysis membranes implanted in rats	14
	DNA microarray	Adaptation to growth in dialysis membranes in rats	15
	DNA microarray	Alteration of lipoprotein expression during host adaptation in mice	16
	Antigenic profiling	Alteration of antigenic profile during host adaptation in mice	17
*Pasteurella multocida*	DNA microarray	Gene expression during growth in blood of infected chickens	18
	DNA microarray	Gene expression during growth in livers of infected chickens	19
*Vibrio cholerae*	DNA microarray	Gene expression during growth in rabbit ileal loops	20
	DNA microarray	Gene expression in rice water stools	21
Interaction with tissue culture cells			
*Chlamydia pneumoniae*	2-dimensional gel electrophoresis	Protein expression during growth in HEp-2 cells in response to interferon-γ	22
*Neisseria meningitidis*	DNA microarray	Interaction with epithelial and endothelial cells	23
	DNA microarray	Interaction with epithelial cells	24

All experiments showed a substantial up-regulation of genes involved in amino acid metabolism, purine biosynthesis, and iron transport and metabolism. Genes in the *ilv* and *pur* operons were consistently up-regulated. Many of the changes also involved up-regulation of genes involved in transport of amino acids and carbohydrates. Indeed, a large number of ABC transport systems were measured as up-regulated. Therefore, in the in vivo environment, whether in rabbit ileal loops, blood, liver, or rice water stools, available nutrients are markedly reduced compared with those in the in vitro medium. Although the current studies have compared gene expression with growth in rich in vitro medium, the major reason for this approach has been the desire to identify potential virulence genes rather that those up-regulated in vivo simply in response to the in vivo nutritional environment. However, many of these genes have been identified by signature-tagged mutagenesis studies as necessary for in vivo survival (25–27).

These studies also found that a number of genes involved in energy metabolism were up-regulated during growth in vivo. Specifically, in each experiment some of the highest up-regulated genes included those encoding particular alternative electron acceptor complexes. In both *V. cholerae*, purified from rice water stools, and *P. multocida*, purified from the blood of chickens, the *nap* (periplasmic nitrate reductase) operon was highly up-regulated. In *V. cholerae*, grown in rabbit ileal loops, the *frd* (fumarate reductase) operon was up-regulated, and in *P. multocida*, purified from the livers of chickens, the *dms* (dimethyl sulfoxide reductase) operon was up-regulated. The appropriate terminal electron acceptor complex is likely determined by the pervading oxygen tension, and oxygen tension differs between different tissues in vivo. Indeed, the growth of *V. cholerae* in rabbit ileal loops and of *P. multocida* in liver indicated up-regulation of a number of genes expected to be regulated by anaerobiosis. Again, these measurements have been compared with growth in vitro in laboratory media so that anaerobiosis is only defined by comparison with the (likely) highly aerobic in vitro environment.

These in vivo experiments have so far shown variable expression of known virulence factors. In *V. cholerae*, in which virulence factors are fairly well defined, a small number of virulence factors were expressed in organisms purified from rice water stools (21), including genes involved in amino acid metabolism, purine metabolism, and the acid tolerance response. None of the genes in the ToxR/TcpP/ToxT virulence gene regulon was identified as differentially expressed in this host niche, which indicates that these genes are transiently expressed and are not necessary as the bacteria are exiting the host. However, a number of virulence genes were expressed in bacteria grown in rabbit ileal loops. Twelve of the top 300 expressed genes in vivo were part of the pathogenesis functional group and included the virulence regulators *tcpP*, *tcpH* and *toxR*, the hemolysin and hemolysin transporter genes *hlyA* and *hlyB*, and the hemagglutinin protease gene *hapR*. For *P. multocida*, one third of the genes identified as virulence genes by signature-tagged mutagenesis (26) were also identified as differentially regulated during growth in the blood of chickens.

Three studies of *Borrelia burgdorferi* have analyzed gene expression during infection (Table). Two of these analyzed whole-genome expression changes during growth in dialysis membranes implanted in rat peritoneal cavities (14,15), and one focused specifically on expression of lipoproteins during growth in mice (16). The gene expression profiles observed differed substantially from those observed for *P. multocida* and *V. cholerae* growing in tissue. Few changes were observed in genes involved in energy metabolism or in amino acid, carbohydrate, and iron transport and metabolism. This finding may be a result of the slow rate of *B. burgdorferi* growth in the mammalian environment. The most notable changes observed in *B. burgdorferi* gene expression involved expression of outer membrane components, particularly lipoproteins (although one study analyzed only lipoprotein genes). Thus, *B. burgdorferi* appears to respond primarily to the host innate or adaptive immune system, or both, resulting in the down-regulation of a large number of surface components, including about 100 lipoproteins.

For many human-specific pathogens, no well-defined animal model exists; conducting gene expression studies during real infections is thus very difficult or impossible. One experimental method that has been used to overcome this problem is analysis of gene expression in response to interaction with host cells. Two studies have analyzed the global transcriptional response of *Neisseria* species to interaction with eukaryotic cells (Table). These studies of *Neisseria meningitidis* and *N. lactamica* compared expression profiles of bacteria in cell culture medium with bacteria in contact with epithelial or endothelial cells (23,24). The gene expression profiles observed in the two studies showed substantial similarity. Similar to the findings from the in vivo studies of *V. cholerae* and *P. multocida* (18–20), many up-regulated genes were identified that were involved in transport and energy metabolism. A range of transporters were up-regulated, especially those involved in amino acid and sulfate transport. Indeed, the sulfate transport system, which is strictly linked to sulfur-containing amino acid metabolism, was up-regulated in the pathogen (*N. meningitidis*) but not in the commensal species (*N. lactamica*), which indicates that this factor may play a role in virulence (24). The other major group of genes that were up-regulated in the bacteria in contact with host cells were those involved in adhesion. Many of these have been previously characterized as virulence genes.

Comparing the in vivo studies with defined in vitro studies may allow deconstruction of the stimuli acting in the in vivo microenvironment. This possibility is a promising aspect of gene expression studies that has not yet been fully explored. For example, in *P. multocida* grown in chickens, the gene expression profile of bacteria within two of three animals was similar to the genes observed to be up-regulated under in vitro iron starvation. Such comparative analyses can expand our understanding of the selective pressures acting on the pathogen during infection. In fact, this first analysis of *P. multocida* indicated that in at least one of the infections, the bacterial gene expression profile differed from that observed under iron-limiting conditions, which suggests that a bacterial response to low iron may occur only in some hosts or at certain stages of infection.

## Proteomic Studies

Proteomics refers to any global analysis of proteins. Proteomics has the potential to show posttranslational modifications, translational regulation, the products of alternative splicing of mRNAs, and selective degradation of proteins, all of which cannot be accounted for when directly measuring mRNA transcript levels.

Although proteomic strategies abound, two main approaches exist for analyzing complex protein mixtures, each of which is quite distinct and possesses subtle advantages and disadvantages. One method relies on the separation of whole proteins by two-dimensional gel electrophoresis (2-DGE) and the subsequent identification of individual proteins through mass spectrometry. The other method, often referred to as multidimensional protein identification technology (MUDPIT), relies on the separation of proteolytic peptides by liquid chromatography and their identification by directly coupled electrospray ionization-tandem mass spectrometry.

A number of technical problems limit the coverage of proteomic analyses. Proteome analysis with 2-DGE often excludes proteins that are large, hydrophobic, or have extremely alkaline isoelectric points. Hydrophobic proteins are often missed because of their insolubility during isoelectric focusing, the problems in extracting hydrophobic peptides from gel matrices, and the difficulty in ionizing hydrophobic peptides for analysis by mass spectrometry (28). Large or basic proteins are often not resolved because they do not enter the isoelectric focusing gradient or do not remain soluble during focusing. The MUDPIT approach overcomes many of the limitations imposed by the solubility difficulties encountered during the isoelectric focusing step of 2-DGE. However, 2-D gels provide a visual reference of protein expression for comparison, while also permitting the experimenter to observe posttranslational modifications and protein cleavage events, which would not be evident by using MUDPIT. In addition, MUDPIT per se does not yield quantitative information (29). 2-DGE and MUDPIT are complementary technologies, and to achieve optimal coverage, both systems should be used (30). However, in contrast to mRNA-based approaches, the current technologies are not capable of elucidating the entire proteome.

Do data obtained by using microarray experiments correlate with proteomic data collected from the same biological system and should we expect them to? In a study performed on yeast in which protein expression with mRNA levels were compared with those obtained by using the serial analysis of gene expression technique, a correlation coefficient of approximately 0.4 was obtained, which indicated that protein expression levels correlated poorly with quantitative mRNA data (31). A more global study, in which mRNA levels obtained by microarray analysis were compared with those obtained with protein expression levels assessed by using a MUDPIT/isotope-coded affinity-tag approach, found that the expression of mRNA and protein sets involved in some biologic pathways were highly correlated while others were not. This finding suggests that posttranscriptional regulation mechanisms were operating in those instances when protein expression levels correlated poorly with quantitative mRNA data (32). Thus, the lack of correlation reported by many researchers (33–36) may result from technical hurdles associated with accurately measuring either mRNA or protein expression levels on a global scale. Nonetheless, if the tools used to measure gene and protein expression are accurate, the expression data should correlate for transcripts and proteins that comprise biologic pathways not subject to posttranscriptional or posttranslational regulation.

Using these proteomic methods to analyze bacterial pathogens has substantial promise, but no example yet exists of global protein expression analysis of a bacterial pathogen growing inside its natural host or in an animal model. This situation has likely occurred because of the technical hurdles associated with separating bacteria from the host tissues and obtaining sufficient material to perform serial analyses required for statistical significance. The problem of contaminating host tissue has probably been overemphasized: small amounts of contaminating host proteins should not compromise the separation and can subsequently be withdrawn from the dataset when in silico searches show a match with the host organism. A far greater problem is obtaining sufficient sample because no techniques are available for signal amplification (as is the case for mRNA expression analysis). Even when enough material can be obtained, this will almost certainly be during the end stages of infection, when the bacterial expression may not differ markedly in quantity from that observed during growth in culture. To analyze global protein expression during the early stages of infection when virulence factors are likely to be expressed, we must await improvements in the technologies involved in separating bacteria from the host and protein expression analysis systems that have improved sensitivity for use with small amounts of sample.

Although no studies of bacterial protein expression inside the host have been published, several investigators have analyzed bacterial protein expression during growth in vitro under conditions that mimic some aspect of infection. These studies have included the response to temperature change, iron-limitation, and the presence of serum proteins (37), nutrient starvation (6), pH stress (38,39), magnesium limitation (40), and biofilm formation (41,42). Other studies have used cell culture systems to more closely mimic the host environment. An analysis of whole cell protein expression of *Chlamydia pneumoniae* (Table) during growth in HEp-2 cells and in response to treatment with interferon-γ was possible after radioactive labeling of the bacteria (22). This analysis indicated up-regulation of a small number of proteins involved in replication, energy metabolism, and peptidoglycan synthesis. An antigen profile analysis of *B. burgdorferi* allowed changes in the antigenic proteins expressed during growth in mice to be compared with changes during growth in in vitro laboratory medium (Table). This analysis allowed for the semiquantitative measurement of *B. burgdorferi* antigen expression in different mouse tissues and showed the differential expression of some known surface proteins (17) However, this analysis falls short of a whole-genome study because it can only measure antigenic proteins soluble in Triton X-114. As technical hurdles are overcome, whole-cell protein expression analysis of bacterial pathogens growing inside the host is poised to provide substantial insight into the mechanisms of bacterial pathogenesis.

## Conclusion

Techniques are now available to begin to meaningfully analyze bacterial expression during growth within eukaryotic hosts, and such studies will transform our understanding of the molecular mechanisms of pathogenesis. Although technical problems remain (such as how to cope with the limited amount of material present during infection and how to purify the pathogens from the eukaryotic host), methods are rapidly being developed to overcome or circumvent these problems. In fact, the necessary further advances will likely be gradual improvements in current technologies rather than new technologies. However, the new challenge may well become the analysis of the large datasets that are generated and the seamless integration with other genomic, proteomic, metabolic pathway, and phenotype data. Integrating these data types will delineate the pathogen's response to the host and help clarify the intricate cross-talk from host to pathogen and the environmental cues and regulatory networks that lead to the expression of bacterial virulence factors. Such a detailed understanding of bacterial pathogens will likely ultimately be available, and this knowledge will facilitate the design of improved vaccines and the rational design of antimicrobial compounds.

This work was funded in part by a project grant from the Australian Research Council.
